# Researches on Meteorology

**Published:** 1848-05

**Authors:** 


*Researches on Meteorology.* By BENNET DOWLER, M. D. (*Reprinted from the New Orleans Medical and Surgical Journal for January,* 1848.

This brochure of twenty-four pages is from the pen of a gentleman known to the profession by researches made some years since, and which are highly creditable to him as a medical observer.

The present off-hand article is confessedly a gift to a "hard-up"

editor, who, no doubt, wanted something from the doctor to fill up the No. "The printer," says Dr. Dowler, "got a leg one day and an arm the next." We congratulate the printer on his good luck; and could wish that every editor, when a little "corner'd," could find an article equally as good as the present one, with as much brains, to say nothing of arms and legs. This, however, is but a prelude to what is to follow, as the author expressly states. We have a bone or two to pick with the doctor, but, still, as they are not very large, we'll let them lie quiet just now, and notice more particularly the subject before us.

We think the word meteorology an unfortunate one—indeed it may be questioned if one out of ten have any distinct idea when the word is spoken; and even on reflection, so diverse are the subjects comprehended under it, that a confusion constantly exists in the mind; and when to this is added the fact, that very little is really known of the science, it being in the main one of modern advancement, it is not at all surprising that clear and definite ideas are not always had on this subject.

To elucidate this point, let us state the various subjects comprehended under this one term, Meteorology, by Prof. Kaemtz, in his work on this subject. Chapt. 1, Treats of the range of temperature in general; Chapt. 2, On winds; Chapt. 3, On aqueous meteors; Chapt. 4, On the distribution of temperature on the surface of the globe; Chapt. 5, On the weight of the atmosphere; Chapt. 6, On the electrical phenomena of the atmosphere; Chapt. 7, Optical phenomena of the atmosphere; Chapt. 8, On the aurora borealis; Chapt. 9, On problematical phenomena.

Surely, to a person unacquainted with the science, here is a melange that might well prove startling; and it must be confessed the science is yet in its very infancy, and will demand for its further development, years of most careful observation by experienced observers.

No one, however, can doubt that meteorology demands the study of every educated medical man. Most certainly atmospheric changes have a decided influence in the production and diffusion of many diseases.

That good old gentleman, Sydenham, long since told us that there was a "medical constitution" of different seasons; and experience every day confirms the truth of the statement. Indeed we have, sometimes, been induced to think that the mortality of many epidemics might be diminished materially, if the medical men would constantly—not now and then—but constantly, observe the meteorological changes of their respective localities, and thus be in some measure, at least, prepared for the character of the epidemic, whatever it may be, that shall present itself. Every practitioner must learn to treat every epidemic as it manifests itself in his locality—not as it is observed by a brother practitioner fifty or sixty miles off. Atmospheric, tellurial influences will modify its type; and the better meteorological observer he is, the sooner, in our humble opinion, will he learn the peculiarities of this type, and the sooner learn how to treat it. Take, for example, the so-called pyrexiæ. How much are they modified, from year to year, in their peculiar characteristics, by the state of the atmosphere in each locality! and how necessarily should the treatment be modified!

With a barometer, a thermometer, and a hygrometer, which can be procured at no very great expense, an individual, with a little industry and patience, may soon learn to be a good observer. The habit of observation will become a pleasure, and, for a medical man, a source of advantage in his daily rounds.

When we think of the number of young men who annually go out from the great medical schools of the country to establish themselves in various places over the vast area of the United States, reaching, in our conquests, to the Pacific, and down even to the Halls of the Montezumas, we cannot but see how much might be done for science—for humanity, if but one half of those who thus go forth yearly would put into practice meteorological observations. Let these observations be regularly printed—thus forming a mass of facts in meteorology—and what incalculable advantage would ensue!

We reserve any particular remarks on the brochure before us for another occasion. †

